# Comparison of Preloaded Bougie versus Standard Bougie Technique for Endotracheal Intubation in a Cadaveric Model

**DOI:** 10.5811/westjem.2015.4.22857

**Published:** 2015-06-23

**Authors:** Jay B. Baker, Kevin F. Maskell, Aaron G. Matlock, Ryan M. Walsh, Carl G. Skinner

**Affiliations:** Madigan Army Medical Center, Department of Emergency Medicine, Dupont, Washington

## Abstract

**Introduction:**

We compared intubating with a preloaded bougie (PB) against standard bougie technique in terms of success rates, time to successful intubation and provider preference on a cadaveric airway model.

**Methods:**

In this prospective, crossover study, healthcare providers intubated a cadaver using the PB technique and the standard bougie technique. Participants were randomly assigned to start with either technique. Following standardized training and practice, procedural success and time for each technique was recorded for each participant. Subsequently, participants were asked to rate their perceived ease of intubation on a visual analogue scale of 1 to 10 (1=difficult and 10=easy) and to select which technique they preferred.

**Results:**

47 participants with variable experience intubating were enrolled at an emergency medicine intern airway course. The success rate of all groups for both techniques was equal (95.7%). The range of times to completion for the standard bougie technique was 16.0–70.2 seconds, with a mean time of 29.7 seconds. The range of times to completion for the PB technique was 15.7–110.9 seconds, with a mean time of 29.4 seconds. There was a non-significant difference of 0.3 seconds (95% confidence interval −2.8 to 3.4 seconds) between the two techniques. Participants rated the relative ease of intubation as 7.3/10 for the standard technique and 7.6/10 for the preloaded technique (p=0.53, 95% confidence interval of the difference −0.97 to 0.50). Thirty of 47 participants subjectively preferred the PB technique (p=0.039).

**Conclusion:**

There was no significant difference in success or time to intubation between standard bougie and PB techniques. The majority of participants in this study preferred the PB technique. Until a clear and clinically significant difference is found between these techniques, emergency airway operators should feel confident in using the technique with which they are most comfortable.

## INTRODUCTION

### Background

Airway management is an essential skill in emergency medicine (EM), and the emergency practitioner must manage all airway emergencies in the critically ill patient. Endotracheal intubation is a key component of emergency airway management. When a difficult airway is encountered, the emergency practitioner must be familiar with multiple intubation techniques, including the use of airway adjuncts.

Endotracheal tube (ETT) introducer is a general term used to describe several similar devices, made from resin-coated, braided polyester, that are used as adjuncts to emergency intubation. These devices are also referred to as “gum elastic bougies” or “bougies.” This article will use “bougie” to refer to ETT introducers generally. In the adult configuration, bougies are typically 60cm in length and 5mm in diameter with an angled tip.^1^ Smaller versions are available for pediatric populations. Bougies are used to assist in the placement of an ETT when glottic visualization is suboptimal, or when other patient factors make orotracheal intubation difficult. Bougies have been shown to be particularly effective when a Cormack-Lehane Grade III view is encountered (epiglottis-only view), or when factors such as obesity, limited cervical mobility or upper airway distortion are present.^2^–^5^ Success rates with bougies for difficult orotracheal intubation have been reported in the range of 74–99%.^3^,^5^–^7^

The procedure for bougie-assisted orotracheal intubation begins with the operator obtaining the best-possible view of the glottic structures with a laryngoscope. This may be accomplished with direct or video laryngoscopy.^8^ The successful use of a bougie has been described with blind digital intubation as well.^9^ The bougie is then passed below the epiglottis, with the angled tip oriented anteriorly, into the glottic opening and confirmed visually as the bougie passes through the vocal cords. Confirmation of blind tracheal placement of the bougie is accomplished by feeling vibrations as the angled tip passes over the tracheal rings and by resistance to further insertion at a depth of 24 to 40cm. Once tracheal placement of the bougie has been confirmed, the ETT is advanced over the bougie into the trachea while the operator seeks to maintain the best-possible laryngoscopic view.^1^,^10^–^11^

**Figure 1:**
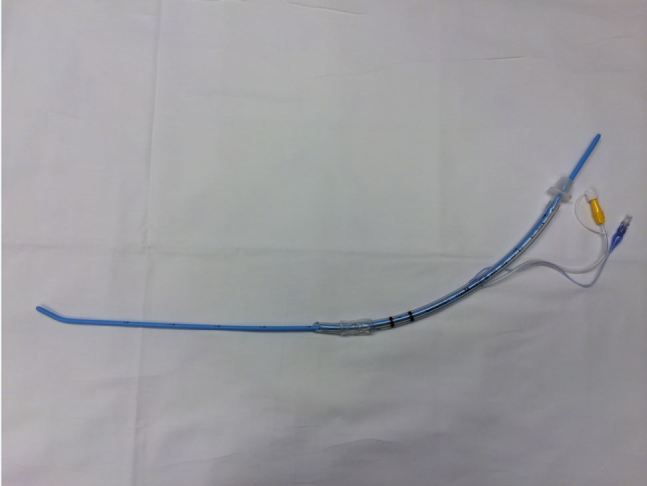
Endotracheal tube preloaded on a bougie.

At our institution, we have observed several instances in which an ETT was “preloaded” onto a bougie by the operator prior to beginning rapid sequence intubation and before initiating laryngoscopy (Figure 1). This is contrary to the instructions of standard emergency airway texts, which describe placing the bougie into the trachea first, then cannulating the ETT over the distal end of the bougie.^1^,^10^–^11^ Typically this step is accomplished by an assistant while the intubator maintains glottic visualization.

### Importance

**Figure 2:**
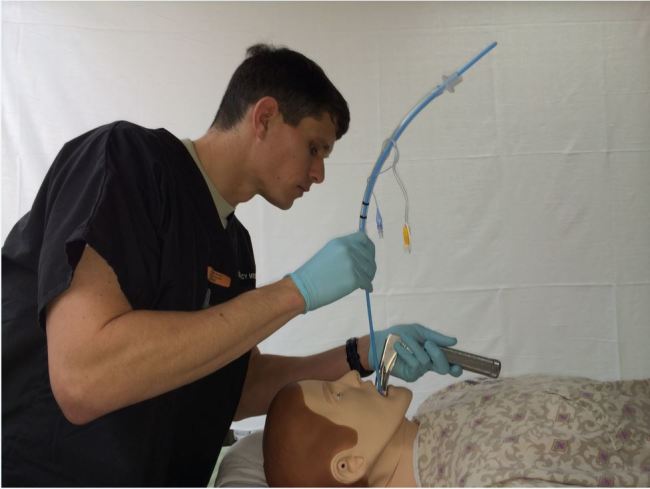
Emergency intubator demonstrating preloaded bougie technique on a mannequin.

The preloaded bougie (PB) technique has been described in multiple online educational forums but has not been formally described in the peer-reviewed literature.^12^–^13^ In the PB technique, the ETT is pre-positioned over the bougie prior to inserting it through the vocal cords. The tip of the ETT is secured in place by the operator’s right hand as the tip of the bougie is placed into the glottis (Figure 2). Following bougie placement through the vocal cords, the already-cannulated ETT is advanced through the vocal cords into the trachea. Thus, a step is eliminated. An assistant is recommended to secure the bougie end after it passes through the vocal cords so that the intubator can maintain a laryngoscopic view with their left hand while sliding the ETT over the bougie with their right hand.

While the use of video laryngoscopy is rapidly growing in the United States, it is not always successful. The bougie will likely continue to be used as an adjunct for difficult airways, either with video laryngoscopy or direct laryngoscopy when video laryngoscopy is unsuccessful or unavailable for any number of reasons. Thus, it is important to identify optimal approaches to using the bougie successfully.

### Goals of This Investigation

In this study, we used a human cadaveric airway model to compare success rates and times to successful intubation between the standard method of bougie-assisted intubation and the novel PB technique. Fresh-frozen cadavers have been shown to have greater airway fidelity when compared to a mannequin model.^14^

## METHODS

### Study Design

This study was a prospective cross-over design using a single study cohort of 47 EM residents (postgraduate years [PGY] 1–3), medical students, EM physician assistants (EMPA) and staff emergency physicians who volunteered to participate. All study participants attended a two-day emergency airway course for EM interns, either as students or instructors. Baseline demographic data was obtained and participants received standardized instruction in intubating with a bougie. Prior to initiation of the study, the local institutional review committee granted this project exemption from review.

### Interventions

Two techniques were taught to each study participant, hereafter referred to as the “intubator.” Standard bougie-assisted (SB) intubation was instructed as follows: a) The bougie was placed through the vocal cords by the intubator after visualizing them with a direct laryngoscope; b) Upon verbal command by the intubator, an assistant placed a 7.5 ETT over the bougie while the intubator continued to hold the bougie in place; c) The intubator then slid the ETT over the bougie, passing it through the vocal cords while maintaining the laryngoscope in place. The assistant secured the end of the bougie until the intubator re-took control and removed it, leaving the ETT in place.

The PB technique was instructed as follows: a) Prior to initiating the procedure, a 7.5 ETT was placed over the end of the bougie up to its 30cm mark with the intubator instructed to hold the bougie below this mark; b) The intubator visualized the vocal cords with direct laryngoscopy then placed the bougie tip through them, while securing the ETT and bougie simultaneously with their intubating hand. After passing the ETT through the vocal cords, the intubator directed the assistant to secure the bougie end; c) While maintaining the laryngoscope in place, the intubator then slid the ETT over the bougie through the vocal cords and then removed the bougie, leaving the ETT in place.

### Methods and Measurements

Two human fresh frozen cadavers were used in order to maximize the number of study participants. Two researchers conducted standardized training with study participants, and an additional two researchers kept time. Both research teams followed the same standards for training and keeping time. Following standardized instruction, each participant was given one attempt to practice each technique before being evaluated. Participants were randomly assigned to start with either technique and consented for participation.

For both techniques, we sought to replicate actual intubating conditions, beginning at the point that the intubator judged the patient to be optimally sedated and paralyzed. The PB technique began with the ETT already in place over the bougie, as we determined that a reasonable intubator wouldn’t paralyze the patient until this was ready, as part of their pre-intubation preparation. The SB technique, on the other hand, began without the ETT in place, just as in real life.

The intubator called “ready” to begin the timer and a bag-valve mask (BVM) was removed from the cadaver’s face. The procedure was judged complete only after the bougie was removed from the ETT and the air cuff was inflated, at which point the timer was stopped. An intubation attempt lasting more than 300 seconds or a non-tracheal intubation was predetermined to be noted as a failed intubation. After intubation, a study investigator used direct laryngoscopy to verify placement of the ETT. After completion of both attempts, participants were asked to report their training level, prior intubation experience including prior experience with a bougie, their perceived ease of intubation on a visual analogue scale of 1 to 10 (1=difficult and 10=easy), and to select which technique they preferred (SB or PB). They were also asked to make a brief comment on the reason for selecting SB vs. PB.

### Outcomes

The primary endpoint of our investigation was time to intubation. Based on previous studies of intubation we anticipated an average time of intubation of approximately 30 seconds.^16^ Using a two-group t*-*test, we determined that a sample size of 24 subjects would allow us to detect a difference of 5s between groups, assuming α=0.05 and a power of 0.80. Secondary endpoints were success rate of intubation, subjective rating by intubators of perceived ease for each intubation technique, and preference for intubation technique. Additionally, each participant was asked to record comments regarding their preference.

### Analysis

We collated, organized, and analyzed data using a Microsoft Excel spreadsheet. A paired t-test was used to determine if there was a difference in time to intubation between the standard bougie technique versus using the PB technique. We used an unpaired t-test to determine the difference for rating ease of use. The sign test was used to determine significance of the stated provider preference.

## RESULTS

### Characteristics of Study Subjects

Forty-seven participants performed intubation using both techniques. The study enrolled 10 medical students, four EMPAs, 19 EM PGY-1 residents, four EM PGY-2 residents, seven EM PGY-3 residents, and three emergency physicians. There was a broad range of reported experience levels (Table). Eighteen participants reported prior experience using the SB technique, whereas only four reported prior experience with the PB technique.

### Main Results

Forty-seven intubations were attempted for each technique. There were two failures for each technique with success rates of 95.7% for both. The failures were by two PGY-1s and one EMPA. One PGY-1 failed both techniques.

The range of times to completion for the standard bougie technique was 16.0–70.2 seconds, with a mean time of 29.4 seconds. The range of times to completion for the PB technique was 15.7–110.9 seconds, with a mean time of 29.7 seconds. There was no statistical difference in time to intubation between the two different methods of intubation in the overall group or any sub-groups (Table).

**Figure 3:**
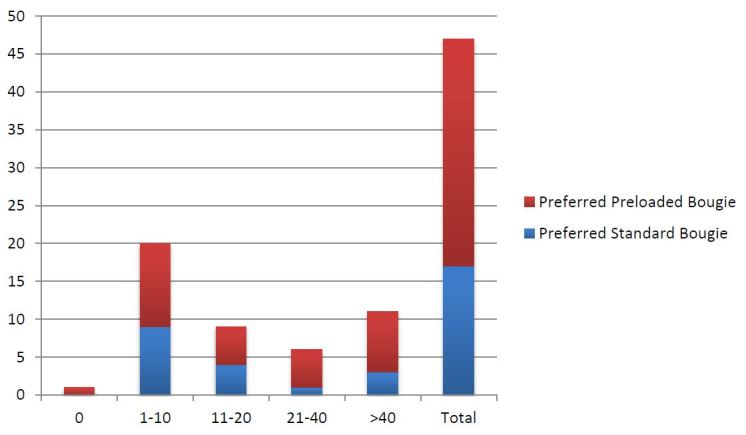
Reported preference for standard vs. preloaded bougie by experience level of intubators, post-study.

**Figure 4:**
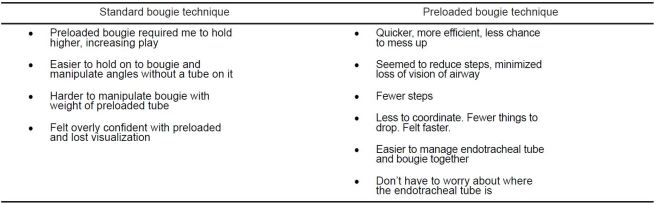
Representative comments regarding preferred technique.

Participants rated the relative ease of intubation as 7.3/10 for the standard technique and 7.6/10 for the preloaded technique (p=0.53, 95% confidence interval of the difference −0.97 to 0.50). Thirty of 47 participants preferred the PB technique over the SB technique (p=0.039). Figure 3 demonstrates selected preferences by experience levels. The majority of participants who preferred the PB technique noted that there were less steps intubating with a PB once the procedure was started. Nearly all the participants who preferred the SB technique cited a decreased sense of control of the bougie using the preloaded method. See Figure 4 for representative comments from each preference group.

## DISCUSSION

In this study, we used a human cadaveric model to compare the previously described standard bougie technique against an unstudied preloaded technique. The preloaded technique may appear to have an advantage, as it begins with the ETT already in place. The standard technique, however, is what is traditionally trained in EM residencies. We aimed to discover if there was any advantage in success or time with either technique.

Our prospective, randomized crossover study found that there was no significant difference in time to successful intubation. These results bore out across all experience levels, with the 95% confidence interval crossing zero in all subgroups (Table).

While the bougie has been in use for decades, we found minimal prior research on alternative techniques. Our results correlate well with other studies on use of the bougie in intubation, with typical times to intubate of approximately 30 seconds.^16^ It seems unlikely that the non-significant differences found here would have any clinical significance in a real-world situation. Our secondary outcome of intubators’ preferences might have a more significant clinical impact by impacting providers’ approaches to intubation with a bougie. The majority of participants in this study group preferred the PB over the standard technique.

Emergent airways are a high stress situation even for experienced providers. Factors such as provider comfort with technique may provide a vital confidence boost. Further, reduction of distractions from concerns rather than positioning the airway device may improve real-world success rates. The most common explanation for preferences by study participants regarding why they preferred one technique or another was related to mechanics. For example, some participants preferred the PB technique for having one less step after paralyzing the patient. Others criticized it for being clumsy, as the added weight of the ETT at the distal end of the bougie interfered with deftly manipulating the bougie tip into the airway. During the course of the study, several participants discovered variations that enabled increased individual comfort. For example, several participants held the ETT closer to their hand earlier in the procedure to increase dexterity. Further study and experiences with alternate techniques may produce additional improvements in technique.

## LIMITATIONS

This research was conducted in conjunction with an airway training course, potentially leading to a bias in perceived ease and success as later participants gained additional practice through the course. The study design essentially controls each participant against him or herself. Further, prior research has demonstrated that the bougie can be effectively taught in a brief time frame.^15^ There remains a potential for bias from participants having recently learned or practiced one method or the other during the concurrent airway course. In addition, these participants were self-selected by virtue of their course attendance to be highly motivated to manage advanced airways. This may limit generalizability to typical airway managers.

Due to limited availability and expense, the same two cadavers were used throughout this study. As a result, each cadaver experienced approximately 100 separate intubation attempts between set up, training, and evaluation attempts, with at least as many additional laryngoscopy attempts to verify ETT placement. Combined with the cadavers’ decreased tissue elasticity, the repeated attempts led to an increasingly well-worn tract along the airway, potentially allowing for increased speed and ease for later participants.

While many experience levels were represented in the study group, the distribution of study participants was skewed toward inexperienced intubators. This was unavoidable due to the setting in which the study was conducted, i.e., an emergency airway course for PGY-1 EM residents. The predominance of inexperienced intubators may have affected the results, as the less experienced groups demonstrated slightly higher times with the preloaded technique. A significant difference might have been found with a larger group of experienced intubators. Additionally, it is possible that the limited diversity of training impacted success rates or introduced bias in terms of participants’ preferences.

It is not clear how the use of video laryngoscopy would have affected the results as only direct laryngoscopy was studied. However, we posit that the differences in success and time between the two techniques would have been minimal with video laryngoscopy as well. Last, the bougie is typically discussed and used as an adjunct for difficult airways. The additional mechanical challenges presented by difficult airway anatomy might make differences in time to intubate more apparent. Preferences might change with the additional stress of a real-world, difficult airway.

## CONCLUSION

There was no significant difference in success or time to intubation between standard bougie and PB techniques. The majority of participants in this study preferred the PB technique. Until a clear and clinically significant difference is found between these techniques, emergency airway operators should feel confident in using the technique with which they are most comfortable, whether it is the standard bougie or PB technique.

## Figures and Tables

**Table:** Mean times for intubation using standard bougie versus preloaded bougie techniques.

Experience level of study participants, by number of intubations	Number of participants	Standard bougie technique, mean (SD)*	Preloaded bougie technique, mean (SD)*	Mean difference (95% CI)
Total	47	29.4 (10.8)	29.7 (16.8)	−0.3 (−6.1,5.5)
>40	11	22.8 (4.94)	19.9 (4.64)	2.8 (−1.4,7.1)
21–40	6	32.7 (16.8)	32.3 (22.97)	0.4 (−25.5,26.3)
11–20	9	33.5 (15.1)	37.1 (28.7)	−3.6 (−26.5,19.3)
1–10	20	29.6 (7.6)	31.1 (10.2)	−1.4 (−7.2,4.3)
0	1	39.2	26.4	N/A

* Mean time to intubation in seconds.
